# Targeting radioresistant breast cancer cells by single agent CHK1 inhibitor via enhancing replication stress

**DOI:** 10.18632/oncotarget.9156

**Published:** 2016-05-04

**Authors:** Yao Zhang, Jinzhi Lai, Zhanwen Du, Jinnan Gao, Shuming Yang, Shashank Gorityala, Xiahui Xiong, Ou Deng, Zhefu Ma, Chunhong Yan, Gonzalo Susana, Yan Xu, Junran Zhang

**Affiliations:** ^1^ Department of Radiation Oncology, School of Medicine, Case Western Reserve University, Cleveland, OH 44106, USA; ^2^ Department of Breast Surgery, Shanxi Academy of Medical Sciences, The Affiliated Shanxi Dayi Hospital of Shanxi Medical University, Shanxi, 030032, PR China; ^3^ Case Comprehensive Cancer Center, School of Medicine, Case Western Reserve University, Cleveland, OH 44106, USA; ^4^ Department of Chemistry, Cleveland State University, Cleveland, OH 44115, USA; ^5^ Department of Breast Surgery, The First Affiliated Hospital of Sun Yat-Sen University, Guangzhou, 510080, PR China; ^6^ Augusta University, Augusta, 30912, GA, USA; ^7^ Department of Biochemistry and Molecular Biology, St. Louis University School of Medicine, St. Louis, MO 63104, USA

**Keywords:** double strand break repair, homologous recombination, replication stress, CHK1 inhibitor, radioresistance

## Abstract

Radiotherapy (RT) remains a standard therapeutic modality for breast cancer patients. However, intrinsic or acquired resistance limits the efficacy of RT. Here, we demonstrate that CHK1 inhibitor AZD7762 alone significantly inhibited the growth of radioresistant breast cancer cells (RBCC). Given the critical role of ATR/CHK1 signaling in suppressing oncogene-induced replication stress (RS), we hypothesize that CHK1 inhibition leads to the specific killing for RBCC due to its abrogation in the suppression of RS induced by oncogenes. In agreement, the expression of oncogenes c-Myc/CDC25A/c-Src/H-ras/E2F1 and DNA damage response (DDR) proteins ATR/CHK1/BRCA1/CtIP were elevated in RBCC. AZD7762 exposure led to significantly higher levels of RS in RBCC, compared to the parental cells. The mechanisms by which CHK1 inhibition led to specific increase of RS in RBCC were related to the interruptions in the replication fork dynamics and the homologous recombination (HR). In summary, RBCC activate oncogenic pathways and thus depend upon mechanisms controlled by CHK1 signaling to maintain RS under control for survival. Our study provided the first example where upregulating RS by CHK1 inhibitor contributes to the specific killing of RBCC, and highlight the importance of the CHK1 as a potential target for treatment of radioresistant cancer cells.

## INTRODUCTION

Radiotherapy (RT) is an effective and commonly employed treatment in the management of more than half of human malignancies, and remains a standard therapeutic modality for breast cancer patients. However, tumors can be intrinsically resistant to RT or develop adaptive response and become resistant. Thus, the curative potential of RT is limited by the radioresistance of the tumor cells. The challenges in breast cancer management are to determine predictive factors that could help to define the subgroups of patients for whom aggressive local therapeutic option is not needed due to their intrinsic resistance, and determine the subgroups of patients who will really benefit from new treatment strategies after failure of RT.

Ionizing radiation (IR) kills cells via causing multiple forms of DNA damage. DNA double strand breaks (DSBs) represent the most dangerous type of DNA damage and is a determining factor of cellular radiosensitivity [1]. DSBs can also be caused by other sources, such as environmental mutagens, chemotherapeutic drugs and any situations causing replication stress (RS) that is defined as slowing or stalling of replication fork progression and/or subsequent fork collapse. One major source causing RS is oncogene expression [2, 3]. DNA damage response (DDR) prevents the cells from lethality due to the damaged DNA via activation of cell cycle checkpoints, promotion of DNA repair, alteration of transcription and triggering apoptosis [4, 5]. Ataxia telangiectasia, and rad3-related (ATR) kinase and its downstream factor CHK1 are core elements of the DDR during replication stress. CHK1 is phosphorylated on serine 317 and serine 345, respectively, by ATR, and these sites are required for the ability of CHK1 to amplify the signal by phosphorylating several additional targets [6, 7]. ATR/CHK1 signaling is important for the cells survival in response to DNA damage agents that cause RS.

ATR/CHK1 is also essential for cell proliferation or viability in the absence of exogenous DNA damage [5, 8–11]. CHK1 promotes replication and transformation in an animal model by limiting oncogene-induced replication stress [12]. In this context, the generation of DNA damage, particularly DSBs induced by oncogenic stress, is suppressed by CHK1 in order to provide the advantage of cell survival. Molecular mechanisms by which ATR/CHK1 maintain viability of cells and suppress oncogene-induced transformation in the absence of exogenous DNA damage are not fully understood. The role of ATR/CHK1 in inhibiting abnormal initiation and elongation of DNA replication [13–15] and maintaining the stability of replication forks [16–18] and promoting replication fork restart could be important mechanisms [19]. Moreover. The role of ATR/CHK1 in homologous recombination (HR) could be also involved since HR is one of important mechanism that repair RS-induced DSBs [20–22]. Thus, ATR/CHK1 signaling is not only critical for the cell survival in the presence of exogenous DNA damage but also essential for cell survival in the absence of exogenous DNA damage, particularly during tumor development.

CHK1 inhibitors have been developed for clinical use, principally with the idea that they would be used to enhance killing of tumor cells by cytotoxic drugs or by radiation, via blocking cell cycle checkpoints, especially in p53 deficient cells [23–26]. Recent studies strongly suggest that sensitization activity of CHK1 inhibitor to IR and/or chemotherapeutic drugs is through a variety of mechanisms, such as inhibition of HR and/or interruption of replication fork stability [27–29]. In addition, although a previous report indicated that CHK1 inhibitor, as a single agent, has none or minimal role in antitumor activity [30], emerging data revealed that CHK1 inhibitor alone can specifically kill some tumor cells [31, 32]. However, the molecular mechanisms controlling the anti-tumor activity of CHK1 inhibitor have not been identified.

The goal of our study is to define the differences of DDR between radiosensitive cells and radioresistant breast cancer cells (RBCC), and to seek for a better regimen targeting the radioresistance. Here, we report that oncogene proteins c-Myc/CDC25A/c-Src/H-ras/E2F1 and DDR proteins ATR/CHK1/BRCA1/CtIP are highly expressed in RBCC. CHK1 inhibition specifically targets RBCC via enhancing RS levels. Our studies, for the first time, apply the concept that increase RS by CHK1 inhibition can target RBCC. Our findings may be more broadly applicable for targeting cancers with similar characteristic as RBCC by CHK1 inhibitors.

## RESULTS

### CHK1 inhibitor, as a single agent, significantly suppresses the growth of cancer cells but fails to sensitize RBCC to IR

**Figure 1:**
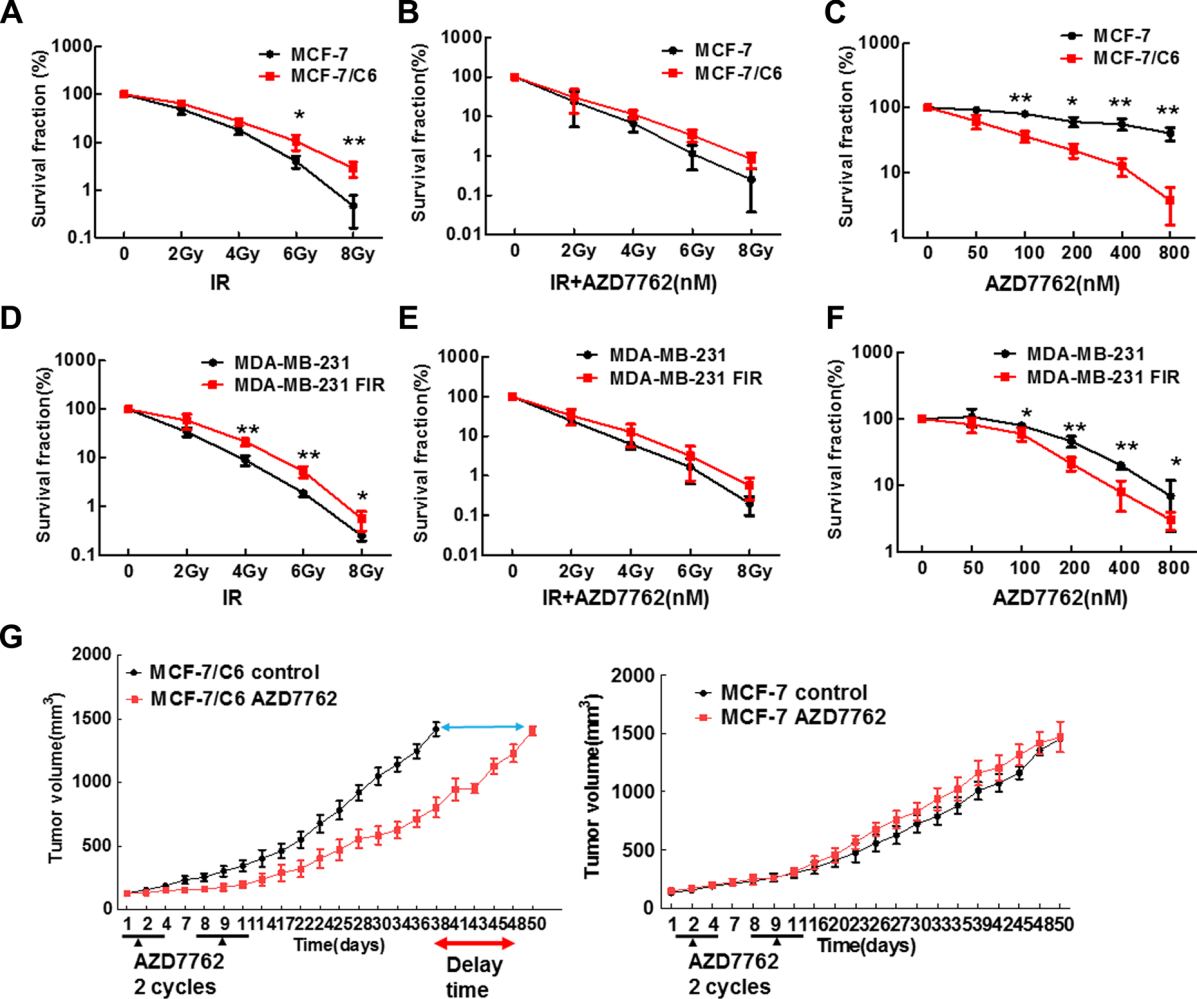
CHK1 inhibitor AZD7762 alone caused more cytotoxicity to RBCC but failed to sensitize RBCC to IR (**A**) Clonogenic survival following IR. Survival experiments were repeated three times and the error bars in the graphs depicting the SD. Values marked with asterisks are significantly different (*T*-test, *p < 0.05, **p < 0.01). (**B**) CHK1 inhibitor (CHK1) failed to sensitize the MCF-7/C6 cells to IR. The cells were treated with AZD7762 (100 nM), then radiated 1 hr later. 24 hr after IR, the drug was removed from medium. (**C**) CHK1 inhibitor AZD7762 alone has more cytotoxicity to MCF-7/C6 cells compared to its own parental cells. Error bars represent the SD of three independent experiments (*T*-test, *p < 0.05, **p < 0.01). (**D–F**) CHK1 inhibitor AZD7762 alone induce more cytotoxicity in MDA-MB-231 FIR cells but AZD7762 failed to sensitize MDA-MB-231 FIR to IR. The methods and statistical analysis are the same as described in A–C. (**G**) Athymic nude mice bearing established MCF-7 or MCF-7/C6 tumors were treated with AZD7762 (25 mg/kg) 2 cycles of therapy 3 days a week (arrows). AZD7762 treatment led to the tumor growth delay with MCF-7/C6 xenografts relative to tumors without treatment (*T*-test, *p < 0.001)*.

Given that CHK1 inhibitor has been reported to sensitize the advanced pancreatic cancer cells to IR [28], CHK1 inhibition could sensitize the RBCC to IR. In order to test this hypothesis, human breast cancer cells MCF-7 and MDA-MB-231, and their corresponding IR -selected radioresistant cells (MCF-7/C6 and MDA-MB-231 FIR) were used [33, 34]. MCF-7 and MDA-MB-231 cells carry wild type p53 and mutant p53, respectively. We first detected the radio-sensitization activity of CHK1 inhibitor AZD7762 using colony formation assay. As we expected, MCF-7/C6 cells are more resistant to IR, compared to their own parental cells (Figure 1A). However, surprisingly, AZD7762 failed to sensitize MCF-7/C6 cells to IR (Figure 1B). Strikingly, we found that CHK1 inhibitor alone caused a dramatic suppression on cell growth in MCF-7/C6 compared to MCF-7 cells (Figure 1C). This result was further confirmed by a second CHK1 inhibitor LY2603618 (Figure S1A). Through measuring sub-G1 cells by flow cytometry, we determined the status of apoptosis. In both MCF-7 and MCF-7/C6, a pattern can be seen where there is a shift in the percentage of sub-G1 cells from viable to apoptosis as the dose escalates (Figure S2A, S2B). However, the percentage of sub-G1 phase cells is significantly higher in MCF-7/C6 cells, compared to control cells at the same conditions. This result was further confirmed by detecting the most optimal biomarkers of apoptosis, such as cleaved caspase 7, 9 and cleaved PARP proteins (Figure S2C). Thus, apoptosis is involved in cell killing induced by CHK1 inhibition. However, we cannot exclude the possibility that other mechanisms of cell death are also involved, such as mitotic catastrophe.

The result that CHK1 inhibitor alone caused a dramatic suppression on MCF-7/C6 cells was further supported by the second cell line MDA-MB-231 FIR (Figure 1D–1F) but the effect of CHK1 inhibition on cell growth is much less significant in MDA-MB-231 FIR (Figure 1F), compared to MCF-7/C6 cells (Figure 1C). Next, in order to further confirm the antitumor activity of CHK1 inhibitor in RBCC *in vivo*, we determined the efficacy of AZD7762 using tumor xenograft models. Groups of tumor-bearing mice were given CHK1 inhibitor or DMSO i.p. daily for 3 days. Two cycles of CHK1 inhibitor were given. For MCF-7/C6 xenografts, the data was analyzed as the time for the tumor volume to reach 1500 mm^3^ (*p* < 0.0001) (Figure 1G, left panel). Clearly, there is a significant delay of tumor growth in the group with inhibitor treatment. Thus, the tumors growth is suppressed when CHK1 inhibitor is administrated in radioresistant MCF-7/C6 xenograft. In contrast, for MCF-7 xenograft, the time for tumor volume to reach 1500 mm^3^ is similar in the group with or without treatment (Figure 1G, right panel). Taken together, our results described in Figure 1 suggest that CHK1 inhibitor AZD7762, as a single agent, can significantly block the tumor growth of RBCC in both *in vitro* and *in vivo* assays.

### Increased expression of oncogene and DDR proteins are induced in RBCC

**Figure 2:**
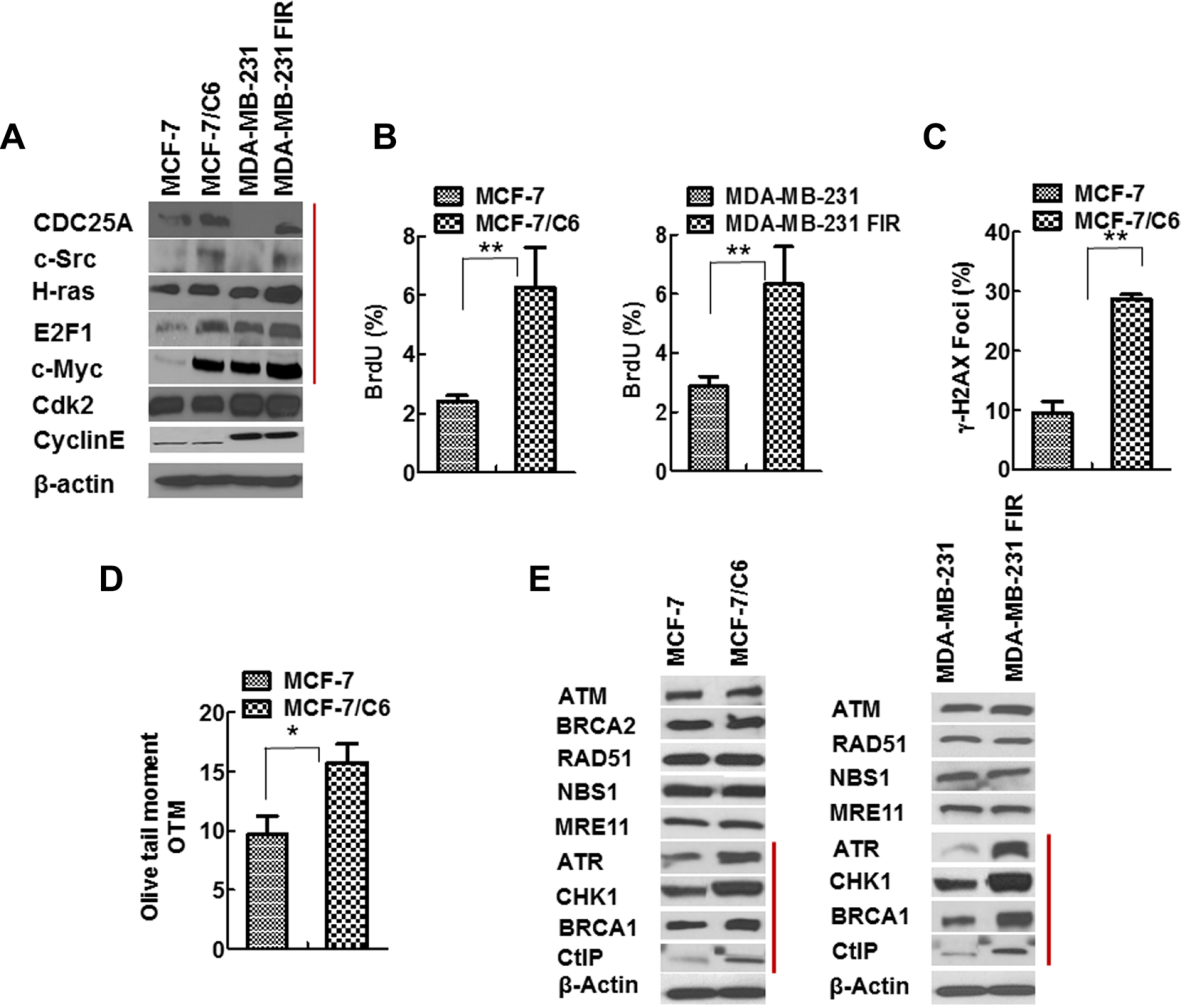
Increased expression of oncogenes and elevated RS in RBCC (**A**) The oncogenes c-Myc/Cdc25A/c-Src/H-ras/E2F1 were induced in RBCC. (**B**) Higher levels of ssDNA accumulation in RBCC. The protocol for ssDNA detection has been described in previous publications [36, 37]. In brief, the cells were grown in the presence of bromodeoxyuridine (BrdU; 10 μg/ml; Invitrogen) for 24 h. After fixation, the cells were blocked and stained with anti-BrdU mouse monoclonal antibody clone B44 (BD Biosciences, San Jose, CA) antibody. Then, the samples are incubated with an Alexa Fluor 488-conjugated goat anti-mouse antibody. Cells were scored positive when 10 nuclear foci were visible. The percentages of cells with BrdU foci are indicated. Error bars indicate SD from three independent experiments (*T*-test, **p < 0.01). (**C**) Higher levels of DSB in RBCC. The percentages of cells with γ-H2AX foci are indicated. In each experiment, 200 nuclei were counted per time. Error bars indicate SD from three independent experiments (*T*-test, **p < 0.01). (**D**) The neutral comet assay of genomic DNA of cells. The results are from three independent experiments (*T*-test, *p < 0.05). (**E**) Increased expression of ATR/CHK1/BRCA1/CtIP in RBCC.

We next ascertained the potential molecular mechanisms by which CHK1 inhibitor specifically targets RBCC. Given that oncogenes can be induced in response to IR [35] and ATR/CHK1 suppresses oncogenic stress [12], we hypothesized that CHK1 inhibition upregulates RS, therefore leading to specific cell killing of RBCC. In order to test this hypothesis, we first determined the expression of oncogene proteins that have been reported to cause RS [2, 3]. Notably, oncogenes c-Myc/CDC25A/c-Src/H-Ras/E2F1 are induced in MCF-7/C6 and MDA-MB-231-FIR cells compared to MCF-7 and MDA-MB-231 cells, respectively, although the magnitude of induction varies (Figure 2A). This result suggests that oncogenic pathways are induced in RBCC.

In support of this hypothesis, we found increased level of RS in RBCC via measurement of single strand DNA (ssDNA) using bromodeoxyuridine (BrdU) labelling (Figure 2B, Figure S3A). This assay is based on the observation that the nucleotide base analogue BrdU is recognized by an anti-BrdU antibody when incorporated into ssDNA but not DSBs [36, 37]. In response to RS, DSBs are often generated due to replication fork collapse. Correspondingly, we observed an increase in the proportion of cells positive for γ-H2AX foci, a marker of DSBs, in MCF-7/C6 cells, compared to MCF-7 cells (Figure 2C, Figure S3B). To further verify that the increased γ-H2AX foci result from an accumulation of DSBs not ssDNA, we next performed comet assay under neutral conditions, which detects DSBs and not ssDNA [38]. Olive tail moment is increased in MCF-7/C6 cells compared to MCF-7 cells (*p* < 0.05) (Figure 2D), indicating that RBCC exhibit accumulation of DNA DSBs. Yet, RBCC are able to proliferate *in vitro* and form tumors *in vivo*, suggesting that these cells have mechanisms in place to cope with RS. In support of the hypothesis that CHK1 is part of the coping mechanism that inhibits oncogene-induced replication stress, the expression of ATR/CHK1 were elevated in MCF-7/C6 and MDA-MB-231 FIR cells compared to parental cells (Figure 2E). In addition, the increased expression of HR proteins BRCA1 and CtIP were also observed in RBCC (Figure 2E). These results described in Figure 2 suggested that the oncogenes c-Myc/CDC25A/c-Src/H-Ras/E2F1 that can cause replication stress and the DDR proteins ATR/CHK1/BRCA1/CtIP that can promote HR are highly expressed in RBCC. In addition, basal level of RS increased in RBCC cells.

### CHK1 inhibition upregulates RS, especially in RBCC

**Figure 3:**
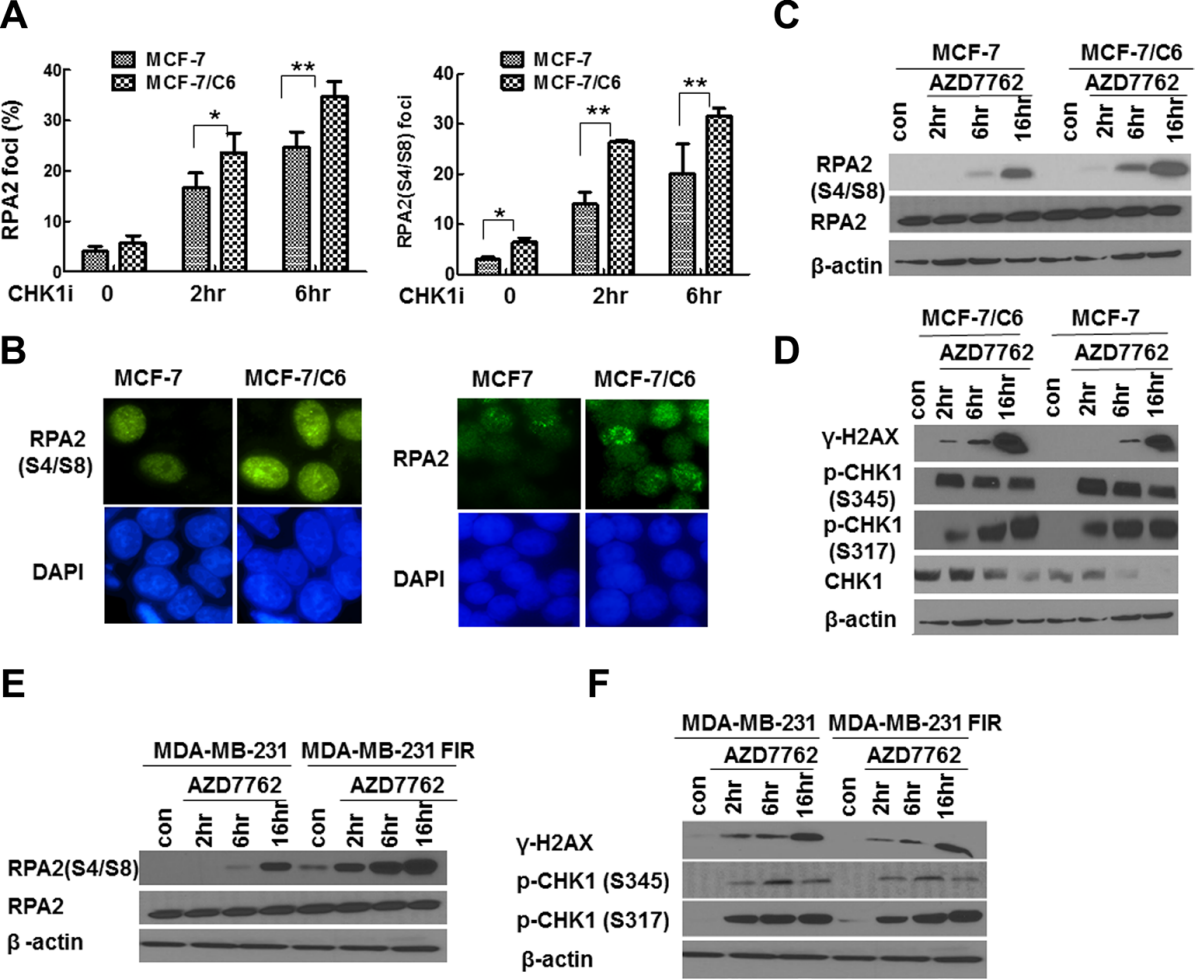
CHK1 inhibition led to the more significant increase in RS in RBCC (**A**) Increased proportion of cells with foci of RPA2 or phosphorylated RPA2 (RPA2 S4/S8) in MCF-7/C6 cells. Data shown are averages from three independent experiments. Error bars represent the SD of three independent experiments (*T*-test, *p < 0.05, **p < 0.01). (**B**) Representative foci of RPA2 (left panel) and RPA2 S4/S8 (right panel) are indicated. (**C**) Expression of RPA2 S4/S8. β-actin or RPA2 are used as loading controls (bottom row). (**D**) Accumulation of DSBs in MCF-7/C6 cells. The measurement of γ-H2AX by immunoblotting using an antibody raised against ser139 phosphorylated of H2AX. The inhibition of CHK1 activity was monitored by the measurement of p-CHK1-345 and p-CHK1-317. (**E**–**F**) CHK1 inhibition led to the more significant increase in RPA2 S4/S8 and there is a space γ-H2AX in MDA-MB-231 FIR cells, compared to control cells.

To determine the extent of RS following CHK1 inhibition, we first analyzed foci of RPA2 and phosphorylated RPA2 (RPA2-P), the markers for RS in response to exogenous DNA damage agents by immunofluorescence staining. A more profound increase in the proportion of cells with RPA2 and RPA2-P foci was observed in MCF-7/C6 cells compared to MCF-7 cells (Figure 3A, 3B). In support of the above hypothesis, the more significant increase of RPA2-P in RBCC cells treated with CHK1 inhibitor was also confirmed by WB (Figure 3C). In addition, CHK1 inhibition led to a more significant increase in γ-H2AX levels in MCF- 7/C6 cells, compared to MCF-7 cells (Figure 3D; Figure S4 left panel). A similar result was also observed in MDA-MB-231 FIR and parental cells (Figure 3E, 3F; Figure S4 right panel). Importantly, CHK1 activity was sufficiently suppressed in our experiments because treatment with CHK1 inhibitor AZD7762 resulted in an increased CHK1 ser345 phosphorylation and reduced global CHK1 protein levels (Figure 3D). It has been demonstrated that CHK1 ser345 phosphorylation is a marker of CHK1 activation [39], which targets CHK1 protein to ubiquitination-dependent degradation [40]. In summary, we conclude that CHK1 inhibition upregulates RS, especially in RBCC.

Since AZD7762 also inhibit CHK2 activity, we next determine how CHK1 or CHK2 knockdown affects RS in RBCC. CHK1 knockdown significantly led to increased γ-H2AX and RPA2-P foci whereas CHK2 knockdown has no detectable effect in RBCC (Figure S5A–S5C), arguing that CHK1 inhibition, instead of CHK2 inhibition by AZD7762, upregulated the extent of RS. These results were further confirmed by western blot (Figure S5D). Thus, the increased levels of RS, evidenced by the accumulation of DSBs and ssDNA are mainly due to the CHK1 inhibition rather than CHK2 inhibition.

### CHK1 inhibition leads to the more significant increase in replication initiation in MCF-7/C6 cells

**Figure 4:**
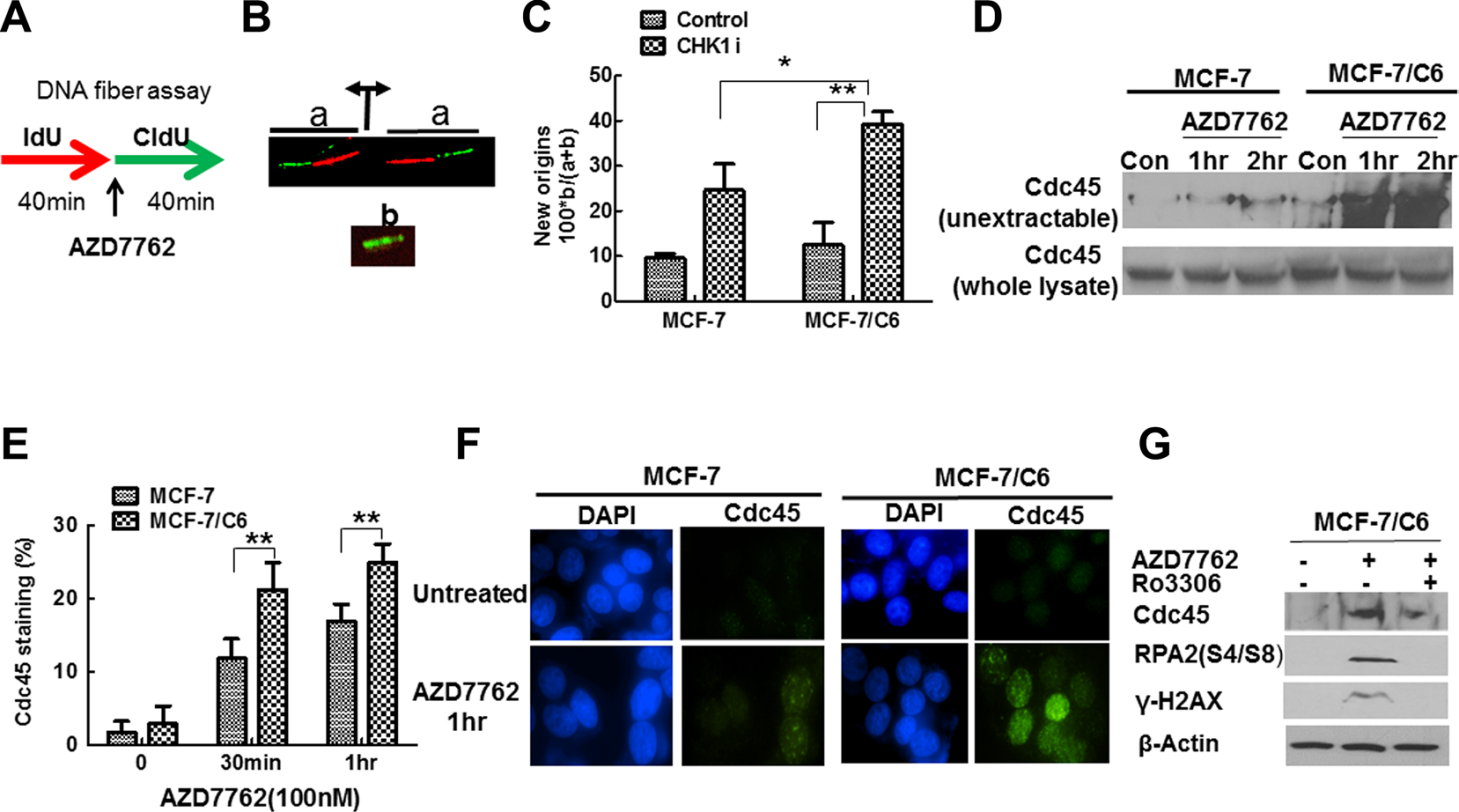
CHK1 inhibition led to the more profound increase in replication initiation in MCF-7/C6 cells (**A**) Schematic of DNA fiber analysis. Green tracks, CldU; red tracks, IdU. (**B**) Schematic drawing and representative images of two replication signals from DNA fibers. At the top, two DNA replication forks moved bidirectionally from an origin (indicated by the diverging black arrows) that was activated before the CIdU pulse. Each fork was labeled with both IdU (red) and CldU (green). At the bottom, the replication bubble resulting from an origin that was activated during the CldU pulse produces a green-only signal. (**C**) Summary of new origins fired during labeling with CldU. The frequency (as a percentage) was calculated as the number of green signals (b in panel B) divided by the total (a + b) of green (b) plus green/red signals (a in panel B). Results are from three independent experiment results (*T*-test, *p < 0.05, **p < 0.01). (**D**) CHK1 inhibition led to increased levels of nonextractable Cdc45 protein in MCF-7/C6 cells. The cells treated with AZD7762 (100 nM) for the indicated time were incubated with extraction buffer for 5 min on ice, and processed for Western blotting (top panel). The whole lysate protein is used as a control (bottom panel). (**E**) Measurement of Cdc45 chromatin loading after preextraction of cells with detergent by immunostaining. Cells presenting with Cdc45 staining were considered positive. The results are from three independent experiments. Error bars represent the SD of three independent experiments (*T*-test, **p < 0.01). (**F**) Representative Cdc45 staining (green) in MCF-7 and MCF-7/C6 cells are presented. Cell nuclei were stained with DAPI (blue). (**G**)The effect of CHK1 inhibition on RS in RBCC depends on Cdk activity. Cdk activity was inhibited by inhibitor Ro3306.

Although the mechanisms by which oncogenes cause RS are not clear, increased origin firing and subsequent nucleoid scarcity are critical reasons in current models [41]. If CHK1 inhibition abrogates the suppression of oncogenic stress, thus increased replication initiation should be observed, especially in radioresistant cells. In order to test our hypothesis, we first determined how CHK1 inhibitor affects DNA replication initiation by analyzing DNA fiber spreads. Cells were sequentially pulse-labeled with IdU and CldU for 40 min each, according to the protocol illustrated in Figure 4A. AZD7762 was added to the cell cultures during the CIdU pulse. IdU and CldU were detected with specific antibodies, in green and red, respectively. Origins of replication that were activated prior to the CldU pulse generated two bidirectional forks, each appearing as a green/red or red/green signal (Figure 4B, signal a). Conversely, new origins that fired during the CldU pulse resulted in a green signal only (Figure 4B, signal b). We quantified the frequency of new origins in untreated and AZD7762-treated cells by dividing the number of green signals (b) by the sum of the green and green/red signals (a + b) (Figure 4B). The percentage of new origins increased when cells were treated with AZD7762 in both parental and MCF-7/C6 cells (Figure 4C), consistent with previous reports that ATR/CHK1 inhibition or depletion increase origin firing in unperturbed cells [42, 43]. However, the magnitude of increase was more significant in MCF-7/C6, compared to parental cells, indicating that CHK1 inhibitor especially targets RBCC.

**Figure 5:**
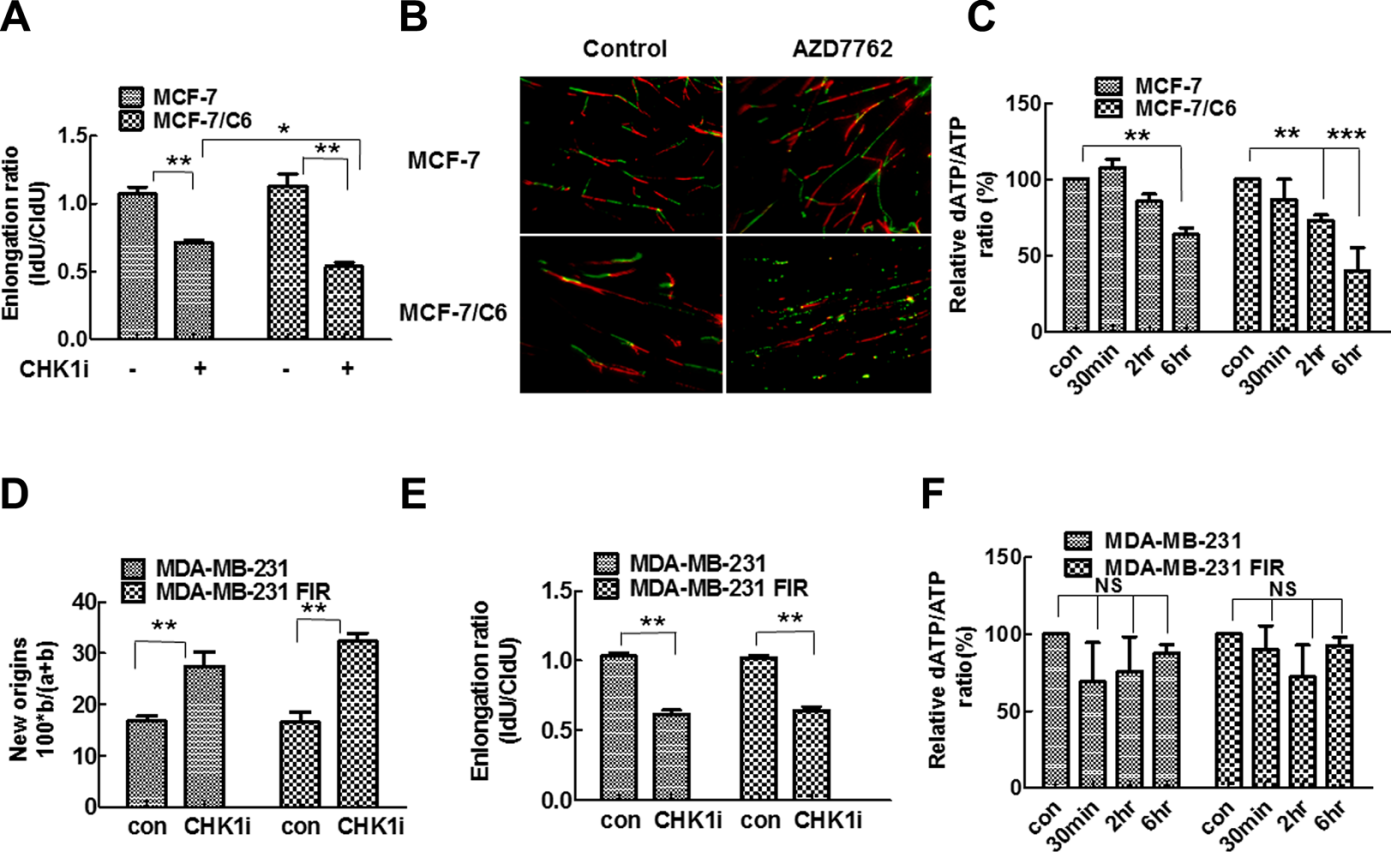
CHK1 inhibition led to the more significant decrease in replication speed and deoxynucleotide supply in MCF-7/C6 cells (**A**) A more significant decrease of replication fork speeds in MCF-7/C6, compared to parental MCF-7 cells following 100 nM AZD7762 treatment. Schematic of DNA fiber analysis is the same as described in Figure 5A. The IdU/CIdU ratio was used to determine elongation. Means and standard deviation (S.D.) of three independent experiments are shown. Values marked with asterisks are significantly different (*T*-test, *p < 0.05, **p < 0.01). (**B**) Representative images of replication tracks from cells treated with or without AZD7762 (100 nm). (**C**) Quantitative determination of ATP and dATP in cell lysates was conducted by LC-MS/MS method. Y axis represents the ratio of dATP/ATP. The details see material and method (*T*-test, **p < 0.01, ***p < 0.001). (**D–F**) CHK1 inhibition had a similar effect on replication dynamics and deoxynucleotide supply in MDA-MB-231 and MDA-MB-231 FIR cells. (D) Summary of new origins fired during labeling with CldU. (E) Replication track length analyzed by DNA fiber spreading. Means and standard deviation (S.D.) The graph is the average of three independent experiments (*T*-test, **p < 0.01). (F) Quantitative determination of ATP and dATP in MDA-MB-231 and MDA-MB-231 FIR cells (NS, no significant difference).

CHK1 is involved in controlling replication initiation via regulating Cdc45 [44], a protein that is implicated in initiation rather than elongation processes. We next measured the amount of Cdc45 in non-extractable chromatin fraction. AZD7762 treatment caused a remarkable increase in the amount of non-extractable Cdc45 protein in MCF-7/C6 cells, compared to MCF-7 cells (Figure 4D). This difference could not be accounted by the differences in Cdc45 levels, which were comparable in the two cell lines (Figure 4D). The effect of CHK1 inhibition on chromatin loading of Cdc45 was further confirmed by IF assay (Figure 4E, 4F). Moreover, Cdk inhibitor Ro3306 treatment abrogated the effect of CHK1 inhibitor on Cdc45 chromatin loading (Figure 4G), which is consistent with a previous report that CHK1 activity on replication initiation is mediated by Cdk activity [13]. Last, Cdk inhibition by Ro3306 prevented accumulation of γ-H2AX and RPA2-P in response to CHK1 inhibitor AZD7762 in RBCC (Figure 4G), suggesting that the increased initiation of DNA replication likely contributes to DSBs generation seen after CHK1 inhibition in radioresistant breast cancer cells. Cumulatively, the results presented in Figure 5 suggest that CHK1 inhibition leads to a significant increase in Cdc45-mediated replication initiation in RBCC.

### CHK1 inhibition leads to a significant decrease in replication fork speed and deoxynucleotide supply in RBCC

Using DNA fiber assay, we next determined how CHK1 inhibition affects replication fork speed in parental and radioresistant cells. We predicted that CHK1 inhibition would reduce replication fork speed due to the increasing origin firing [45]. The significant decrease in the speed of replication fork progress was observed in both MCF-7 and MCF-7/C6 cells when CHK1 activity is inhibited (Figure 5A, 5B). However, the magnitude of the decrease is more significant in MCF-7/C6 cells compared to MCF-7 cells (Figure 5A). These results show that CHK1 inhibition slows down fork progression while increasing origin firing (replication initiation) in breast cancer cells, and that the effect is more robust in RBCC (Figure 5A, 5B). These data are also consistent with the report that CHK1 promotes replication fork progression by controlling replication origin activity [14]. Although it is not known how the increased origin firing could lead to slow replication fork progression, the imbalance in dNTP pools can cause dysfunctional replication [46, 47]. Thus, we next determined dATP levels by Liquid chromatography-tandem mass spectrometry (LC-MS/MS). Indeed, dATP levels was reduced by 60% following CHK1 inhibitor treatment in MCF-7/C6 cells which is significantly higher than the 36% reduction seen in MCF-7 cells (Figure 5C), indicating that CHK1 inhibition led to the increased deoxynucleotide consumption, especially in RBCC. Interestingly, a similar phenotype was found in MDA-MB-231 pairs (Figure 5D–5F), but with some differences. CHK1 inhibition has an equivalent effect on replication initiation (Figure 5D) and fork speed (Figure 5E) in parental MDA-MB-231 and radioresistant MDA-MB-231 FIR cells. However, CHK1 inhibition failed to cause scarcity of dNTP pool in both MDA-MB-231 and MDA-MB-231 FIR cells (Figure 5F). These results suggest that the increased replication firing may not necessarily lead to depletion of dNTP pool in all types of tumor cells, and also that the mechanisms by which CHK1 inhibition leads to increased levels of RS may be not be limited to regulation in replication initiation and nucleotide pool balance.

### CHK1 inhibition leads to more significant decrease in HR activity in RBCC

**Figure 6:**
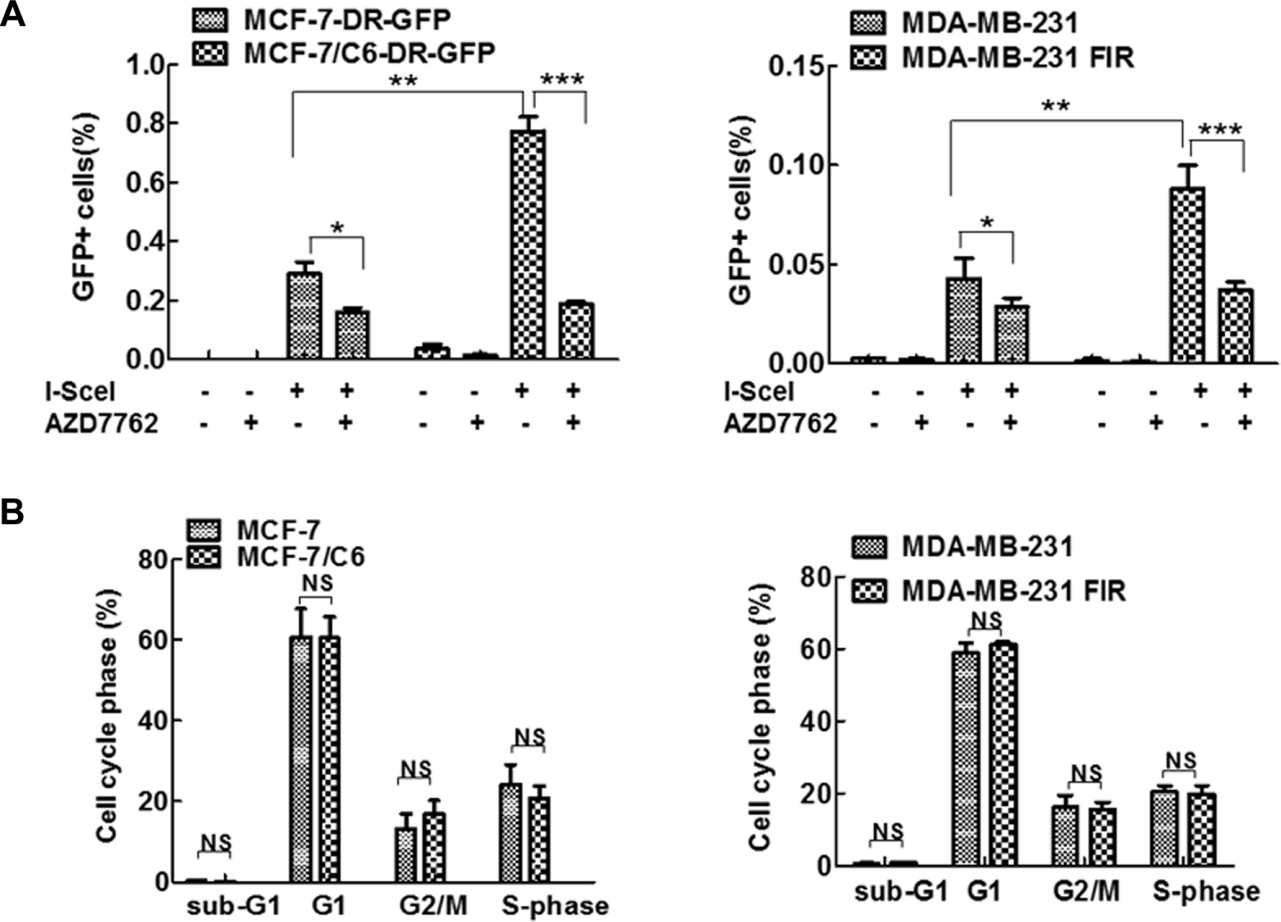
CHK1 inhibition resulted in a more significant decrease in HR in RBCC (**A**) AZD7762 exposure led to decreased HR-mediated repair, particularly in RBCC. HR was detected using chromosomally integrated HR substrate (DR-GFP) which is based on reconstitution of the EGFP (from M. Jasin). HR induced by I-SceI was measured by dual-color flow cytometric detection of GFP-positive cells. In brief, the cells were transfected with I-SceI and then AZD7762 (100 nM) was added to the medium 24 hr after transfection. HR was measured 24 hr after the addition of AZD7762. Error bars represent the SD of three independent experiments (*T*-test, *p < 0.05, **p < 0.01, ***p < 0.001). (**B**) Cell cycle profiles are indicated. Cell cycle was analyzed by flow cytometry. Results are means from three independent experiments. Error bars represent the SD of three independent experiments (NS, no significant difference).

In addition to interruption of replication dynamics, CHK1 inhibition may also impair HR. HR is a mechanism that suppresses RS induced by oncogenes, by promoting the repair of DSBs that result from replication fork collapse. Thus, we next determine the role of CHK1 inhibitor in HR activity in both parental and RBCC by HR reporter *DR-GFP* as described previously [36, 48–51]. In this system, a DSB is generated by expressing the I-SceI endonuclease. Repair of the cleaved I-SceI site by gene conversion-associated HR gives rise to a functional GFP gene when the template used for repair is a truncated GFP fragment located downstream in the plasmid. HR activity is measured by flow cytometric analysis of the number of GFP+ cells following I-SceI expression [51]. The parental MCF-7 cells and MCF-7/C6 cells with chromosome integration of DR-*GFP* were established using a standard method [50]. Using the established system, an increased frequency of HR in MCF-7/C6 and MDA-MB-231 FIR cells was observed, compared to their own parental cells (Figure 6A). The increased HR in RBCC co-related with the increased expression of ATR/CHK1/BRCA1/CtIP since these proteins are important for HR activity [20, 21, 52, 53]. The increased HR activity was not caused by the alteration of the cell cycle because identical cell cycle profiles were observed in radioresistant cells and their corresponding parental cells (Figure 6B). We found that CHK1 inhibition leads to a more significant decrease in HR in RBCC, compared to parental cells. Collectively, these data suggest that CHK1 inhibition results in a significant decrease in HR activity, particular in RBCC cells. This result is consistent with the observation that CHK1 inhibition led to a more profound increase in RS in RBCC (Figure 3). Thus, RBCC cells most likely depend on HR activity for survival because HR is a major mechanism counteracting the DNA damage caused by RS.

## DISCUSSION

**Figure 7:**
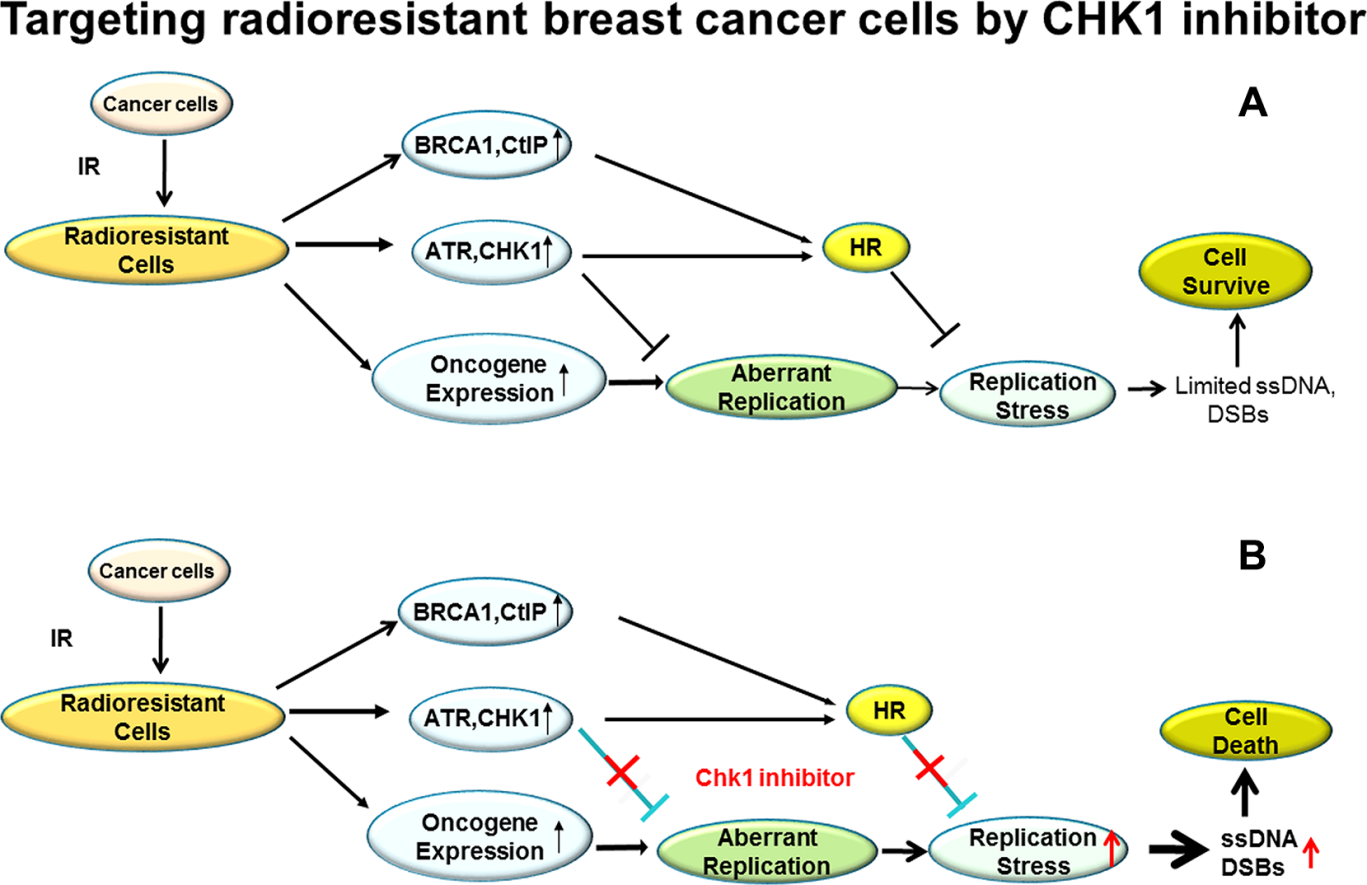
A proposed model for targeting RBCC cells by CHK1 inhibitor via abrogating the suppression in RS induced by oncogenes (**A**) The RBCC express high levels of DDR proteins and oncogene proteins, including ATR/CHK1/BRCA1/CtIP and c-Myc/CDC25A/c-Src/H-ras/E2F1. The increased expression of DDR protein would be an important mechanism suppressing oncogenic stress by inhibiting aberrant replication initiation and promoting HR. Therefore, the damages caused by oncogenic stress in RBCC are minimal and the RBCC with high levels of expression of oncogene proteins survive. (**B**) CHK1 inhibition enhances oncogenic stress by abrogating the suppression of replication initiation and/or interrupting HR activity, which leads to the accumulation of massive ssDNA/DSBs and subsequent death of RBCC.

Chemotherapeutic drugs that as single agents can specifically target radioresistant cancer cells are rarely reported and studied although continuing efforts have been conducted to identify radiosensitizing agents that preferentially sensitize tumor cells to the cytotoxic action of RT. In contrast to the common paradigm that CHK1 inhibitor can be used as a radiosensitizer, in this study we report that CHK1 inhibitor, as a single agent, can specifically target RBCC via regulation of RS (Figure 7), reducing the growth of tumor cells *in vitro* and *in vivo*. Thus, CHK1 inhibition may provide therapeutic opportunities in the radioresistant breast cancer patients.

### Oncogene proteins and DDR proteins are induced in RBCC

Oncogenes c-Myc/CDC25A/c-Src/H-Ras/E2F1 and DDR proteins ATR/CHK1/BRCA1/CtIP are also highly expressed in RBCC (Figure 2). The physiological significance of the increase in the expression of both oncogene proteins and DDR proteins in RBCC is not fully understood. However, according to the oncogene-induced DNA damage model of cancer progression [2, 3], oncogenes generate substantial amounts of RS, which in turn activate the DDR. Activation of DDR is important to limit the expansion of tumor cells with RS [54–56], since RS causes the genomic instability that facilitates the acquisition of secondary hits in the genome that promote malignancy. To minimize the impact of this effect and to maintain the fitness of the cell, the activation of oncogenes is often associated with compensatory molecular changes, processes mediated in part by the ATM and ATR protein kinases [19]. If this is the case, the induced oncogene expression developed during RT could be toxic to cells due to RS increase. However, the increased ATR/CHK1/BRCA1/CtIP expression may constitute a key step to enhance cellular tolerance to oncogenic stress, considering their roles in suppression of abnormal replication initiation and promotion of HR, which are two important mechanisms suppressing oncogenic stress [12, 22]. Therefore, radioresistant cells are selected because they confer a growth advantage by overcoming the toxicity of oncogenic stress via enhancing DDR protein expression during RT therapy.

Currently, considerations for radiotherapy are determined by the clinical factors rather than molecular subtypes and pathways, which might result in the unnecessary treatment for the patients who are intrinsically resistant to IR. Whether higher expression of oncogene proteins c-Myc/CDC25A/c-Src/H-ras/E2F1 and DDR proteins ATR/CHK1/BRCA1/CTIP can be used as indicators for the predication of radioresistance need to be determined in the clinic in the future.

### Induced essentiality and targeting RBCC by CHK1 inhibitor

Several concepts originated in genetics have been applied to cancer therapy. “Synthetic lethality” describes the situation where a defect in one gene or protein is compatible with cell viability but results in cell death when combined (synthesized) with another gene or protein defect. This concept has been practically applied for the treatment of breast cancer patients that are defective in BRCA1/2 with PARP inhibitors [57, 58]. “Induced essentiality” is an extension of synthetic lethality [59] that refers to a new state in which mutation of a gene drives the tumorigenic phenotype of the cells but also has potentially deleterious effects on cell fitness. Activity of a second gene mitigates the deleterious effects of the mutation of the first gene. Thus, the second gene/protein is essential for cell survival. We speculate that the increased DDR protein expression is important for the survival of radioresistant cells where the oncogenes are highly expressed. It is noteworthy that in our study we observed a more effective antitumor activity by CHK1 inhibition in RBCC where the oncogenes are highly expressed (Figures 1, 2). Therefore, it is possible that targeting radioresistant cancer cells with CHK1 inhibitor is an extension of the concept of “Induced Essentiality” to cancer therapy. In support of this concept, ATR/CHK1 pathway inhibition in combination with oncogene expression of H-ras^G12V^ cells elevate H2AX phosphorylation to significantly higher levels than produced in control cells [55], and also ATR/CHK1 inhibitors are highly effective in killing Myc-driven lymphomas [56]. Thus, the greatest effect of CHK1 inhibition in cancer treatment may be achieved in different types of cancer cells with similar characteristic as RBCC, based on the same logic. This hypothesis needs to be intensively tested in future.

Several potential mechanisms could contribute to this specific targeting of RBCC by CHK1 inhibitor. First, the radioresistant cells may rely on ATR/CHK1/BRCA1/CTIP for survival in the absence of exogenous DNA damage due to their critical role in inhibition of replication initiation and/or HR promotion as we discussed above. Second, one of the major differences between radioresistant and parental cells is that RBCC carry a high level of RS (Figure 2). The additional DNA damage, as a result of the increased replication initiation by CHK1 inhibition, may saturate the DSBs repair ability. Under conditions of proficient DNA repair, both radioresistant and parental cells may be able to accomplish full repair. However, if HR is inhibited, the radio-resistant cell's additional burden of lesions saturates the DNA repair capacity and specifically sensitizes the tumor cells relative to the normal. The increased burden of DNA DSBs and the impaired HR due to CHK1 inhibition provide a synthetic lethal interaction that would be selective for RBCC. These differences between parental and RBCC provide a unique opportunity to target RBCC.

The radiosensitization activity of CHK1 inhibitor was observed in previous studies [23, 24, 28], perhaps due to the interruption of G2/M arrest and HR activity [28]. In our study, CHK1 inhibitor failed to sensitize RBCC to IR although HR activity and G2/M arrest in RBCC are abrogated by CHK1 inhibitor (Figure 6, data not shown). These results suggest that the defects on HR and G2/M arrest may not necessarily sensitize RBCC to IR. This result is consistent with the fact that G2/M phase checkpoint plays a minimal role in radio sensitivity [60]. In addition, HR is less important than Non-homologous end joining (NHEJ) for repair of IR-induced DSBs, although this pathway is critical for the repair of DSBs that originate during DNA replication in S phase cells. Thus, it is most likely that regardless of the impairment of HR and G2/M arrest caused by CHK1 inhibition, NHEJ pathway can be still active or upregulated in RBCC cells, which could sufficiently repair DSBs induced by IR. In support of this hypothesis, NHEJ activity is significantly higher in RBCC, compared to parental cells (data not shown). Thus, multiple mechanisms contribute to the radioresistance of RBCC. Blockage of HR and G2/M arrest by CHK1 inhibitor are not sufficient to sensitize these cells to IR.

### CHK1 inhibition increases RS levels in RBCC

Our results suggest that CHK1 inhibitor targets RBCC via regulation of RS caused by oncogenes (Figure 3). The mechanisms by which oncogenes induce RS have not been clearly defined. According to a current model, oncogene-induced RS is a result of hyper-replication, with an increase in replication initiation and the subsequent scarcity of replication factors, such as nucleotides [61]. The cellular availability of nucleotides may not be sufficient to carry out replication under conditions where a massive number of origins are fired simultaneously, thus slowing replication fork. Subsequently, ssDNA and DSBs are induced, and cell death occurs as a result of massive DNA damage. In support of our hypothesis that CHK1 inhibitor kills RBCC via upregulation of oncogenic stress, we find a significant increase in the firing of replication initiation, a decrease in dNTP pool, and a reduction in fork progression in MCF-7/C6 cells, in comparison to parental control cells (Figures 4, 5). However, this may not explain all since CHK1 inhibition enhances cell killing and replication stress in MDA-MB-231 FIR cells compared to MDA-MB-231 cells. However, the effect of CHK1 inhibitor on replication initiation/fork speed and dATP pool is similar in MDA-MB-231 FIR and MDA-MB-231 cells. Since HR activity is also an important mechanism suppressing oncogenic stress the effect of CHK1 inhibition in HR activity may contribute to the specific killing of MDA-MB-231 FIR cells (Figure 6). In our study, only limited oncogene expression was detected (Figure 2A). Given that different oncogenes cause RS via distinct mechanisms, it could be possible that the effect of CHK1 inhibition on replication initiation might depend upon the context of tumor cells.

In conclusion, we propose a model in which CHK1 inhibitor can be used to specifically target RBCC. CHK1 specifically limits RS by inhibiting replication initiation and promoting HR in RBCC. In normal conditions, RBCC survive even with high levels of induced oncogene expression during RT (Figure 7A). However, when CHK1 activity is inhibited, the suppression of replication initiation is abrogated, which leads to increased amounts of DSBs. On the other hand, the decreased HR activity due to CHK1 inhibition could also result in a failure to repair DSBs (Figure 7B). The dual roles of CHK1 inhibitor in interruption of replication initiation and HR contribute to its antitumor activity in RBCC (Figure 7). In our model, targeting RBCC and upregulating RS by CHK1 inhibition appears to be independent of p53 since CHK1 inhibition leads to more cell killing and the more profound increase in RS, compared to their parental cells regardless of the status of p53 (Figures 1, 3). Our result is consistent with a recent publication indicating that oncogenic stress sensitizes murine cancers to hypomorphic suppression of ATR, and the toxic interaction between ATR suppression and oncogenic stress occurred independent of p53 status [54]. However, we cannot exclude the possibility that p53 is involved to certain extent, because a remarkable cell killing by CHK1 inhibitor is observed in wild type p53 expressing cells (MCF-7/C6) in comparison to mutant p53 expressing cells (MDA-MB-231 FIR) (Figure 1).

In summary, our study reveals that upregulation of RS could be a promising strategy targeting radioresistant breast cancer cells by CHK1 inhibition. In addition, it is conceivable that RBCC or any situations harboring same characteristics as RBCC may be prime candidates for treatments utilizing CHK1 inhibition.

## MATERIALS AND METHODS

### Cell lines, infections, transfections and inhibitors

Parental cells and their corresponding radioresistant derivatives were obtained from JianJian Li (University of California Davis). MCF-7 and MCF-7/C6 cells were cultured in Minimum Essential Medium (MEM, lifetechnologies) supplemented with 10% bovine growth serum, 100 U/ml penicillin and 100 μg/ml streptomycin at 37°C, 5% CO_2_. MDA-MB-231 and MDA-MB-231 FIR cells were cultured in Dulbecco's Modified Eagle Medium (DMEM with Low Glucose, HyClone) supplemented with 10% bovine growth serum, 100 U/ml penicillin and 100 μg/ml streptomycin at 37°C, 5% CO_2_. MCF-7/C6 cells with chromosomal integration of the DR-GFP reporter were generated according to a standard protocol. CHK1 and CHK2 short-hairpin RNAs (shRNA) were purchased from Sigma. All DNA plasmid transfections were performed using Lipofectamine 2000 according to the manufacturer's recommendations (Invitrogen, Carlsbad, CA). The CHK1 inhibitors AZD7762 and LY2603618 were purchased from Selleckchem and ApexBio, respectively. The Cdk inhibitor Ro3306 was purchased from Tocris.

### Immunoblotting

The following conditions are used. Anti-BRCA1 (Clone D-9, 1:200, Santa Cruz Technology); Anti-RAD51 (Clone H92; 1:200; Santa Cruz Technology); Anti-BRCA2 (Clone 5.23, 1:500, EMD Millipore); Anti-RAD52 (Clone 5H9, 1:200, GeneTex); anti-RPA2 (Clone NA18, 1:100, Calbiochem/EMD Millipore); Anti-53BP1(Clone 1B9, 1:1000, Novus biologicals); Anti-E2F1(Clone KH95, 1:200, Santa Cruz Technology); Anti-β-Actin (Clone AC-74, 1:10000, Sigma-Aldrich); Anti Mre11 (1:1000, Novus Biologicals); Anti-CHK1 (G-4, 1:500, Cell signaling); Phospho-CHK1 antibody (#2344 CHK1-pSer317,1:500; Cell signaling); Phospho- CHK1 antibody (#133D3 CHK1-pSer345, 1:500, Cell signaling); Anti c-Myc (9E10 sc40, 1:300, Cell signal), Anti E2F1 (clone KH95 sc-251, 1:500, Cell signaling); H-ras(F235 sc-29, 1:50, cell signaling); c-Src(N-16 sc-19, 1:50, cell signaling); Anti Cdc45 (G-12 sc55569, 1:50, Santa Cruz); γ-H2Ax (ser139 JBC301, 1:500, Millipore clone); rabbit polyclonal antibody phosphor RPA32 Ser4/Ser8 (Bethyl, BL647, 1: 1000 dilution), Anti CDC25A (clone DCS-120, 1:100, Thermo scientific); Cdk2 (610146, 1:200, BD Biosciences); cyclin E (sc247, 1:200, Santa Cruz Technology) for western blotting. Secondary antibodies used were goat-anti-mouse IgG–horseradish peroxidase (HRP) conjugated, goat-anti-rabbit IgG–HRP conjugated at 1:1000 dilutions. Radiation was delivered to cultured cells using a cesium-137 gamma ray at a dose rate of 3.1 Gray/min.

### Immunofluorescence analysis

Mouse anti-γ-H2AX (Ser139, clone JBW301, millipore) was used at 1:500 dilution. The monoclonal anti-BrdU antibody (BD Biosciences) was used at 1:200 concentration. Rabbit anti- RPA32 (S4/S8) [A300-245A, BETHYL] were used at 1:500 dilution. For analysis of Cdc45 chromatin staining, a detergent extraction method was employed as described previously (9). Rabbit anti-Cdc45 (H- 300, clone, sc20685, Santa Cruz) was used 1:50 dilution. The secondary antibody, goat–anti-mouse IgG Alexa fluor 594 or FITC-conjugated anti-mouse secondary antibody (sigma) was used at 1:400 dilution. The slides were viewed at 1000 × magnification on an NIKON 90i fluorescence microscope (photometric cooled mono CCD camera).

### Homologous recombination assay

HR was measured in cells according to previous publications [50].

### Cell cycle analysis

Cell Cycle Analysis was conducted as we described previously [50].

### Comet assay

Cells were analyzed by the Comet assay under neutral conditions (Trevigen, Gaithersburg, MD). Comets were analyzed using CometScore software (TriTek, Sumerduck, VA).

### Colony formation assay

Clonogenic analysis was performed as described in reference [50]. Colonies containing > 50 cells were counted.

### DNA fiber assay

DNA fiber assay were performed as published previously [62]. The replication fibers were viewed at 1000× magnification on an NIKON 90i fluorescence microscope (photometric cooled mono CCD camera). Signals were measured by using Image J software (NCI/NIH), with some modifications made specifically to measure DNA fibers.

### LC-MS/MS method for quantitative determination of ATP and dATP in cell lysate

The cells (1 × 10^7^) were suspended in 0.5 ml of 80% methanol aqueous solution. The mixture went through a process of freezing (−80°C) and thawing (room temperature) for 3 times, followed by a sonication for 10 min, and then centrifuged. The supernatant was dried with N2. The residue was dissolved in 400 μl of 10 mM ammonium formate solution, and 100 μl of the solution was used for LC-MS analysis. The separation of analytes was carried out on an XTerra C18 column (2.1 × 100 mm) using a gradient elution from 20% B to 80% B in 12 min at a flow rate of 0.2 ml/min. The mobile phase A was 1 mM tributylamine and 0.25 mM acetic acid aqueous solution, and mobile phase B was 90% methanol with 1 mM tributylamine and 0.25 mM acetic acid. All calibrators, internal standards (ATP-d4 and dATP-13C10,15N5), and QC samples were prepared in 10 mM ammonium formate solution. 10 μl of samples was injected. This method has a linear calibration range of 5.00–500 ng/ml for dATP and 100–50000 ng/ml for ATP. The API 3200 mass spectrometer was operated using ESI–. Quantitation was done by MRM mode with the following parameters: m/z 506 > 273 for ATP, m/z 510 > 159 for ATP-d4, m/z 490 > 159 for dATP, m/z 505 > 159 for dATP-13C10,15N5, dwell time at 50 ms, declustering potential (DP) at −53 V, entrance potential (EP) at −6.0 V, collision energy (CE) at −30 V, collision cell exit potential (CXP) at −6.0 V, curtain gas (CUR) at 25, collision gas (CAD) at 3, ionspray voltage (IS) at −4500V, temperature (TEM) at 600°C, ion source gas 1 (GS1) at 50, ion source gas 2 (GS2) at 50, and resolutions were set at unit for both Q1 and Q3.

### Xenograft studies

Female athymic nude mice, 4–5 weeks of age, bred in Case Western Reserve University, were used for this study. All experiments were carried out under a protocol approved by the National Cancer Institute Animal Care and Use Committee and were in compliance with the Guide for the Care and Use Of Laboratory Animal Resource, (1996) National Research Council. For tumor growth delay studies, Implant the 17β-ESTRADIOL(innovative research of America cat#NE-121 0.72 mg/pellet 90-day release) into the mice on the neck one week prior to cells injection, then 8 × 10^6^ cells were suspended and then injected subcutaneously into flanks. Tumor growth was followed until the diameter of tumor reached 0.6–0.8 cm. At this point animals were randomized into 2 groups (8 mice/group): control and AZD7762. AZD7762 (25 mg/kg) was administered by i.p. injection at 2,3,5 days (once a day) as a cycle for 2 cycles. Students *T*-test was used to calculate the delay of two group and *p*-values for the differences between the various groups.

## SUPPLEMENTARY MATERIALS FIGURES


