# Supplementary material for: Targeting radioresistant breast cancer cells by single agent CHK1 inhibitor via enhancing replication stress
Source: Oncotarget. 2016 May 4;7(23):34688–702. doi: 10.18632/oncotarget.9156 (PMC5085184; doi:10.18632/oncotarget.9156)
Supplement: Supplementary file 1 [file oncotarget-07-34688-s001.pdf]

Targeting radioresistant breast cancer cells by single agent CHK1 inhibitor via enhancing replication stress

Supplementary Materials

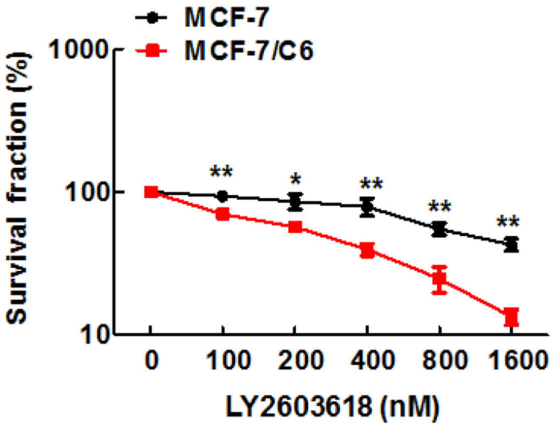

Supplementary Figure S1: CHK1 inhibitor LY2603618 alone has more cytotoxicity to MCF-7/C6 cells compared to parental cells. Clonogenic survival experiments were repeated three times and the error bars in the graphs depict the SD. Values marked with asterisks are significantly different (T-test, *$p < 0.05$, **$p < 0.01$).

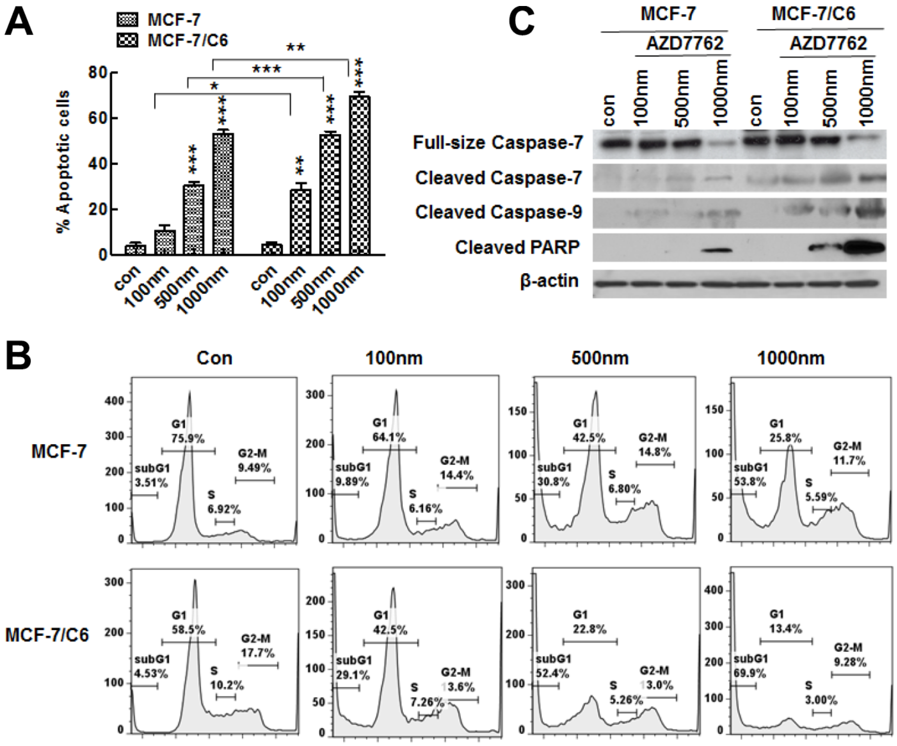

Supplementary Figure S2: CHK1 inhibition promotes apoptosis in beast cancer cells, especially in the RBCC. (A) CHK1 inhibition induced more significant apoptosis in RBCC. The parental cells and RBCC were treated with CHK1 inhibitor AZD7762 at the indicated three concentration for 48 hrs, then fixed and stained with propidium iodide. Flow cytometric analyses were used to profile sub-G1, G1, S and G2-M cell populations. Apoptotic cells were quantified. Error bars represent the SD of three independent experiments (T-test, *$p < 0.05$, **$p < 0.01$, ***$p < 0.001$). (**B**) Representative flow cytometry profiles of apoptosis. (**C**) CHK1 inhibition increases pro-apoptotic activity through cleaved caspase 7, cleaved caspase 9 and cleaved PARP protein activation within 24 hrs of treatment with three different doses of AZD7762. The CHK1 inhibition -induced apoptosis was detected by the indicated biomarkers of apoptosis.

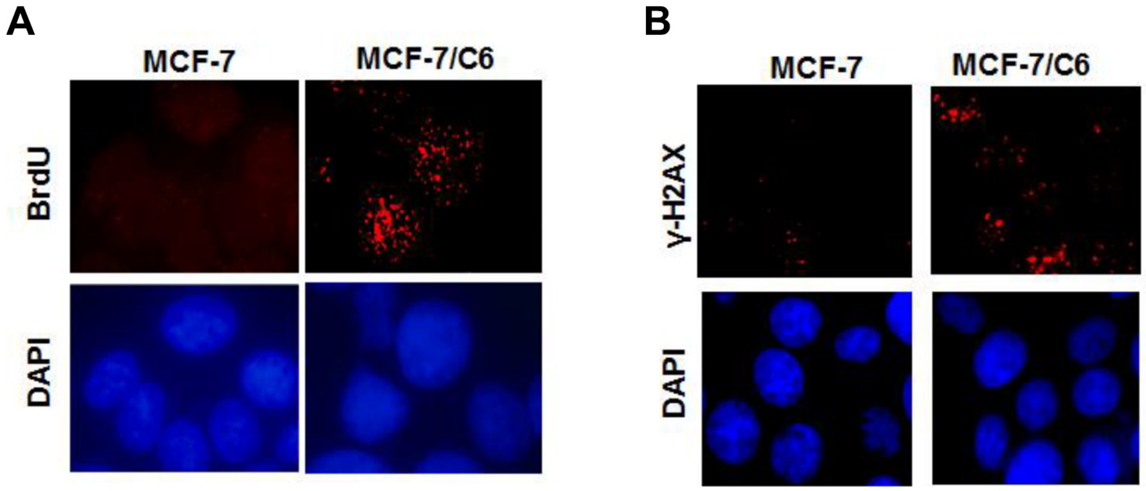

Supplementary Figure S3: Representative ssDNA foci and γ-H2AX foci in MCF-7 and MCF-7/C6 cells.

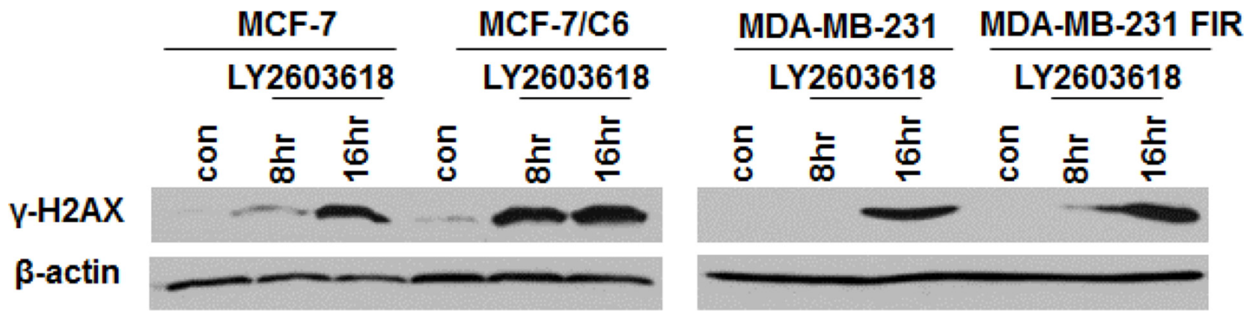

Supplementary Figure S4: CHK1 inhibitor LY2603618 alone causes more significant increase in γ-H2AX levels in RBCC. The measurement of γ-H2AX by immunoblotting using an antibody raised against ser139 phosphorylated of H2AX.

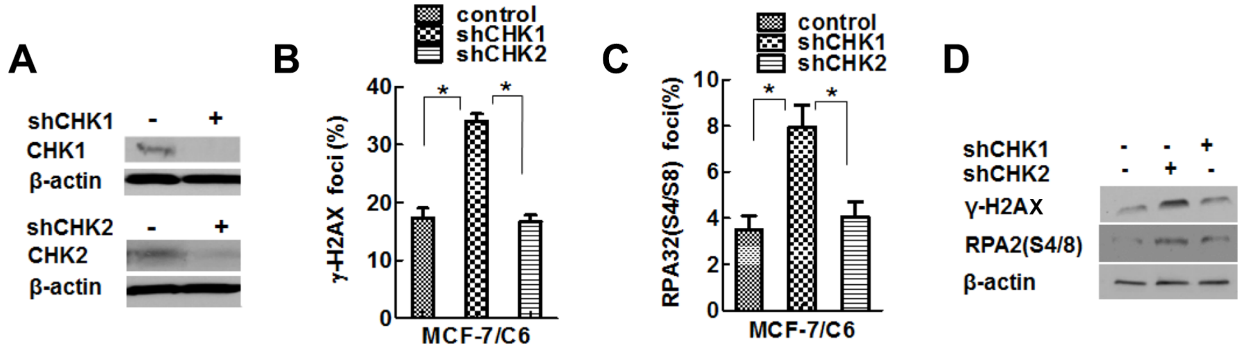

Supplementary Figure S5: Downregulation of CHK1 not CHK2 led to increased levels of RS. (**A**) CHK1 or CHK2 knockdown via shRNA in MCF-7/C6 cells. (**B, C**) Knockdown CHK1 but CHK2 resulted in increased percentage of cells with γ -H2AX and phosphorylated RPA2 (S4/S8) foci. The percentages of cells with γ-H2AX and RPA2 (S4/S8) foci are indicated. Error bars indicate SD from three independent experiments (*T*-test, *$p < 0.05$). (**D**) The accumulation of DSBs and ssDNAs in cells with knockdown of CHK1 but CHK2. The levels of γH2AX and RPA2 (S4/S8) were detected by immunoblotting. β-actin was used as a loading control.
